# Electrophysiological and behavioral responses of *Tamarixia radiata* (Hymenoptera: Eulophidae) to volatiles of nymphal *Diaphorina citri* (Hemiptera: Liviidae)

**DOI:** 10.1093/jisesa/ieae060

**Published:** 2024-05-29

**Authors:** Yan-Mei Liu, Yuan-Yuan Huang, Fei-Feng Wang, Yu-Wei Hu, Zhi-Lin Zhang, Andrew G S Cuthbertson, Bao-Li Qiu, Wen Sang

**Affiliations:** Engineering Research Center of Biological Control, Ministry of Education/College of Plant Protection, South China Agricultural University, Guangzhou 510642, China; Engineering Research Center of Biological Control, Ministry of Education/College of Plant Protection, South China Agricultural University, Guangzhou 510642, China; Guangdong Laboratory for Lingnan Modern Agriculture, Guangzhou 510642, China; Engineering Research Center of Biological Control, Ministry of Education/College of Plant Protection, South China Agricultural University, Guangzhou 510642, China; Guangdong Laboratory for Lingnan Modern Agriculture, Guangzhou 510642, China; National Modern Agricultural Industry Science and Technology Innovation Center of Guangzhou, Guangzhou 510520, China; Hubei Key Laboratory of Quality Control of Characteristic Fruits and Vegetables, College of Life Science and Technology, Hubei Engineering University, Xiaogan 432000, China; Independent Science Advisor, York YO10 5AQ, UK; Guangdong Laboratory for Lingnan Modern Agriculture, Guangzhou 510642, China; Engineering Research Center of Biological Control, Ministry of Education/College of Plant Protection, South China Agricultural University, Guangzhou 510642, China; Guangdong Laboratory for Lingnan Modern Agriculture, Guangzhou 510642, China

**Keywords:** parasitoid choice, olfactory response, host location, electrophysiological activity, *trans-*2-nonenal

## Abstract

Huanglongbing (HLB), a devastating citrus disease caused by *Candidatus* Liberibacter asiaticus, is efficiently vectored by the Asian citrus psyllid, *Diaphorina citri* Kuwayama (Hemiptera: Liviidae). *Tamarixia radiata* (Waterston) plays a crucial role as an ectoparasitoid, preying on *D. citri* nymphs. By collecting and identifying headspace volatiles from fifth instar nymphs of *D. citri* using a gas chromatograph–mass spectrometer (GC–MS), we obtained a collection of 9 volatile compounds. These compounds were subsequently chosen to investigate the electrophysiological and behavioral responses of female *T. radiata*. At a concentration of 10 μg/μl, 9 compounds were compared with *cis-*3-hexen-1-ol (control), resulting in *trans-*2-nonenal inducing the highest relative electroantennogram (EAG) value, followed by hexanal, heptanal, *n-*heptadecane, tetradecanal, *n-*tetradecane, *n-*pentadecane, 1-tetradecanol, and 1-dodecanol. The top 5 EAG responses of female *T. radiata* to these compounds were further investigated through EAG dose–response experiments. The results showed positive dose–responses as concentrations increased from 0.01 to 10 μg/μl. In Y-tube olfactometer bioassays, female *T. radiata* exhibited a preference for specific compounds. They were significantly attracted to tetradecanal at a concentration of 10 µg/µl and *trans-*2-nonenal at 0.01 µg/µl, while no significant attraction was observed toward hexanal, heptanal, or *n-*heptadecane. Our report is the first to demonstrate that volatiles produced by *D. citri* nymphs attract *T. radiata*, which suggests that this parasitoid may utilize nymph volatiles to locate its host.

## Graphical Abstract

**Graphical Abstract:**
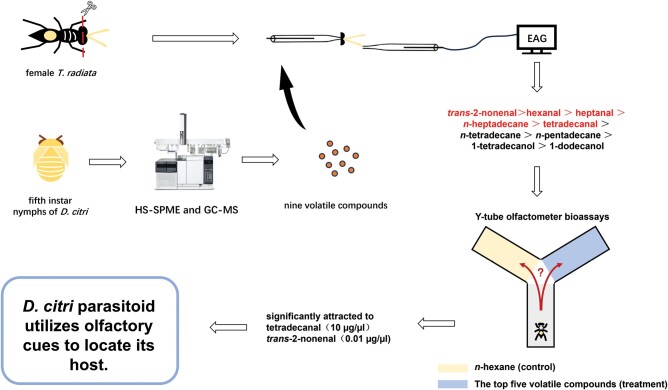

## Introduction

Huanglongbing (HLB) is one of the most destructive diseases of citrus. It leads to the development of bitter, inedible, and misshapen fruits, eventually causing the death of infected trees within 5–10 years (Bové and Barros 2006). This disease is caused by *Candidatus* Liberibacter asiaticus (*C*Las), which can be efficiently transmitted by the insect vector Asian citrus psyllid, *Diaphorina citri* Kuwayama (Hemiptera: Liviidae) (Halbert and Keremane 2004). At present, due to insect vector migration, HLB has spread to many countries including Mexico, Belize, Puerto Rico, Cuba, USA, and China, causing significant economic losses (Hall et al. 2013). Various vector and disease control strategies are being employed worldwide in citrus cultivation, including biological control with the ectoparasitoid *Tamarixia radiata* (Hymenoptera: Eulophidae) (Rosas et al. 2013, Hajeri et al. 2014, Hawkings et al. 2014, Miranda et al. 2016). This parasitoid can both feed on and parasitize *D. citri* nymphs, effectively reducing *D. citri* populations (Ramiaranjatovo et al. 2023).

An adult female *T. radiata* typically lays eggs beneath a *D. citri* nymph. Upon hatching, the parasitoid larvae immediately feed on the hemolymph of the *D. citri* nymph at their attachment site. After eclosion, the adult parasitoid emerges from the anterior region of the *D. citri* mummy (Milosavljević et al. 2017). Many studies have focused on wasp-rearing methodologies, distribution, parasitization success, and developmental biology (Skelley and Hoy 2004, Hoddle et al. 2014, Chen et al. 2017, Gebiola et al. 2018, Li et al. 2018). However, limited information addresses how *T. radiata* searches for its insect hosts. Understanding this information is crucial for comprehending how *T. radiata* locates its hosts and could also contribute to enhancing the efficacy of biological control. When *D. citri* feeds on citrus plants infected with *C*Las, the plant-derived volatile methyl salicylate is produced and released. This compound can attract psyllids as well as *T. radiata* (Mann et al. 2010, 2014, Martini et al. 2014). Consequently, it can be speculated that certain plant volatiles, such as methyl salicylate, serve as chemical cues used by the wasps for host-habitat location. A previous study revealed that not all nymphal stages were suitable for parasitism, with female parasitoids exhibiting significant attraction toward third, fourth, and fifth instar nymphs and showing a preference for later instar nymphal stages of *D. citri* for oviposition (Li et al. 2018). As a result, there is no doubt that other olfactory cues from the insect host are involved in the host location process of *T. radiata*. In the present study, we tested the volatile profile of fifth instar nymphs of *D. citri* using electroantennogram (EAG) measurements. Subsequently, the main identified compounds were subjected to Y-tube olfactometer behavioral analysis to address this hypothesis.

## Materials and Methods

### Plants and Insects

The plants used in the study were seedlings of *Murraya paniculata* (L.). These were cultivated within glasshouses at South China Agricultural University (SCAU). They were maintained within plastic containers (30 cm × 30 cm) under the following conditions: temperatures fluctuating between 20 °C and 35 °C and relative humidity (RH) of between 56% and 85%. The seedlings received regular irrigation and fertilization as required. *D. citri* were collected from wild *M. paniculata* plants surrounding the campus of SCAU and subsequently then cultured on the *M. paniculata* plants within the laboratory under the following conditions: 26 ± 1 °C, 80 ± 10% RH, and a 14:10 h Light:Dark (L:D) photoperiod. To stimulate oviposition of *D. citri* fresh *M. paniculata* flushes were provided. *T. radiata* adults, also collected from *M. paniculata* plants surrounding the campus of SCAU, were introduced to infest the fifth instar nymphs of *D. citri* under the same controlled environmental conditions. Upon emergence, the resulting *T. radiata* adults were reared in glass tubes with a 20% sugar solution as a nutritional supplement.

### Insect Volatile Collection

Volatiles from fifth instar nymphs of *D. citri* were collected using a headspace solid-phase micro-extraction (HS-SPME) collection system. Five hundred nymphs were placed into a sample bottle (treatment group), and the stainless steel needle of the SPME extraction head was suspended into the bottle for 1 h at room temperature. Blank controls are empty bottles without nymphs. The fiber coating is polydimethylsiloxane and made by Supelco Company (Bellefonte, PA, USA). The dimension and cap type of glass bottle is 500 ml and silicone plug. All trials were conducted with 3 repetitions.

### GC–MS

Headspace extracts were analyzed using Agilent Technologies 7890b gas chromatograph (GC) linked to a 5975C mass spectrometer (MS). The apparatus used for separation was as follows: DB-5MS column, Agilent, 30 mm length, 0.25 mm ID, and 0.25-μm film thickness. In addition, the oven temperature of the column was maintained at 35 °C for 2 min before being raised to 100 °C at 5 °C/min, and then finally raised to 250 °C at 10 °C/min; this final temperature was then held for 20 min. The ionization mode was set at electron ionization (EI) = 70 eV. Overall, the area scanned was 29–500 amu. After the working parameters of the instrument were set and the working conditions of the instrument were ready, the SPME was inserted into the GC injection port, and the extraction head was fixed in the injection mouth at 250 °C for 5 min by rapidly pushing and turning the SMPE push rod. The push rod was then taken out. The carrier gas was helium and the flow rate was 3.0 ml/min. The preliminary identity of the compounds produced were then determined by comparison with the available standard spectrum library (NIST 11.0).

### Chemicals

Synthetic chemical compounds identified by GC–MS were procured from different companies. Hexanal (98%) (CAS RN: 66-25-1), heptanal (95%) (CAS RN: 111-71-7), and *trans-*2-nonenal (95%) (CAS RN: 18829-56-6) were obtained from Tokyo chemical industry (Tokyo, Japan). Tetradecanal (96%) (CAS RN: 124-25-4) was purchased from Aladdin. *n-*Tetradecane (99.4%) (CAS RN: 629-59-4), *n-*pentadecane (99.5%) (CAS RN: 629-62-9), and *n-*heptadecane (99.8%) (CAS RN: 629-78-7) were acquired from Dr. Ehrenstorfer GmbH (Augsburg, Germany). 1-Dodecanol (99%) (CAS RN: 112-53-8) and 1-tetradecanol (98%) (CAS RN: 112-72-1) were purchased from ANPEL Laboratory Technologies Inc. (Shanghai, China). Furthermore, the *cis-*3-hexen-1-ol (CAS RN: 928-96-1), used as a reference control in the EAG tests, was purchased from Tokyo chemical industry (Tokyo, Japan).

### Electrophysiological Experiments

Antennal responses of *T. radiata* to chemical compounds were detected using GC-EAD with an Agilent Technologies 7890B GC coupled with an EAG detector (Syntech, Kirchzarten, Germany). To analyze the response of the parasitoid antenna to specific reference compounds, commercial compounds were subjected to EAG dose–response tests at a concentration of 10 µg/µl. The units for the EAG value is mV. The 5 compounds that elicited the strongest EAG response for *D. citri* nymphal volatiles were then selected for further investigation in an EAG dose–response test. The dissected antenna (with half of the head) from female *T. radiata* that were 4–9 days old were fastened with saline solution onto 2 sharpened glass capillaries (Syntech, Kirchzarten, Germany). The dose–response test involved a series of concentrations of the tested compounds dissolved in *n-*hexane, ranging from 0.01, 0.1, 1 to 10 µg/µl, for the comparative EAG response tests. Prior to testing, 20 µl of each chemical solution was applied to a piece of filter paper strip (2 cm × 4 cm). The solvent was allowed to evaporate from the filter paper for 30 s before it was placed into a glass tube. A continuous airflow of 0.2 L/min provided in 0.5 s puffs was maintained via a stimulus controller. For each compound tested, there were 6 replicates from 6 female *T. radiata*. The interstimulus interval between concentrations for the dose–response was 45 s. The EAG value (%) was determined in comparison to that received for *cis*-3-hexen-1-ol. The control of *n-*hexane and its resulting EAG value was determined at both beginning and end of the trial. The EAG relative value was determined using the following equation:


$$EAGrelativevalue=\text{(}R_{1}-R_{3}\text{)}/\text{(}R_{2}-R_{3}\text{)}$$

where *R*_1_ represents the EAG value of the tested standard substance, *R*_2_ denotes the EAG value of *cis*-3-hexen-1-ol, and *R*_3_ signifies the EAG value of *n-*hexane.

### Olfactometer Behavior Assays

The 5 compounds that elicited the strongest EAG responses were chosen to determine their impact on adult female *T. radiata* via a Y-tube olfactometer. The 5 compounds were dissolved in *n-*hexane in order to create the following concentrations for use in the Y-tube response tests: 0.01, 0.1, 1, and 10 µg/µl. The olfactometer consisted of the same apparatus as described by Liu et al. (2019); a Y-shaped glass tube with a 0.8 cm inner diameter with the base and the 2 arms of the Y-tube measuring 6 cm in length. Within the Y-tube arms, the airflow was maintained at a constant rate of 80 ml/min. Room air was then pumped via activated charcoal and a 500 ml Pyrex Erlenmeyer flask containing distilled water using vacuum pressure. The environmental conditions were carefully regulated at 26 ± 1 °C and a RH of 60 ± 10%, with a red-light intensity of approximately 12 lux maintained above the Y-tube equipment. The behavioral experiments were conducted between the hours of 12:00 PM and 18:00 PM.

Adult female *T. radiata* ranging between 4 and 9 days old were individually placed into the base of the Y-tube and consequently observed for 5 min. *T. radiata* odor choice was recorded when a parasitoid entered a given arm of the olfactometer and remained there for at least 1 min. The odor source (each at 4 concentrations: 0.01, 0.1, 1, and 10 µg/µl) was then placed on a piece of filter paper inside a glass bottle. For the testing treatments, 20 µl of each compound at each concentration was applied to a piece of filter paper strip, allowing the solvent (*n-*hexane) to evaporate for 30 s. This was then connected to one of the 2 arms of the olfactometer. The different compounds at their different concentrations were then tested separately. Meanwhile, a control consisting of a filter paper strip treated with 20 μl of *n-*hexane was connected to the other arm of the olfactometer. At the initiation of each bioassay a given odor source was randomly assigned to an arm of the olfactometer. This was then changed after every 10 adults to eliminate bias. Before initiating all trials, the experimental wasps were exposed to clean air vs. clean air in the olfactometer in order to ensure there was no positional bias. No significant preference in either arm was noted via this test. Following each treatment test all experimental equipment was thoroughly cleaned using a warm deionized water and soap solution before being placed in a drying cupboard at 75 °C for 12 h. Each odor treatment trial consisted of 30 adult female *T. radiata*.

### Data Analysis

The data from the bioassays were analyzed using analysis of variance via the software SPSS 17.0 (SPSS Inc., Chicago, IL, USA). *χ*^2^ tests were used for analyzing the olfactometer behavioral assay data. One-way ANOVA was used for analyzing the EAG data with means obtained compared via the multiple comparison test at *P* < 0.05.

## Results

### Qualitative Identification of Nymph Volatiles

In this experiment, we used retention time and mass spectrometry to identify the volatiles according to the GC–MS results. In comparison to blank control, the treatment group has shown an increase in the diversity of volatile compounds. Specifically, the compositions of volatiles from nymphs consisted of 9 compounds (hexanal, heptanal, *trans-*2-nonenal, tetradecanal, *n-*tetradecane, *n-*pentadecane, *n-*heptadecane, 1-dodecanol, and 1-tetradecanol) (Table 1).

**Table 1.** Volatile compounds of nymphal *Diaphorina citri*

Compounds	Retention time (min)
Hexanal	18.180
Heptanal	8.043
*trans-*2-nonenal	16.730
Tetradecanal	16.965
1-Tetradecanol	19.085
*n-*Tetradecane	11.625
*n-*Pentadecane	21.165
*n-*Heptadecane	22.570
1-Dodecanol	22.240

The retention time of the compound in the GC–MS is used to identify the substance.

### EAG Analysis

**Fig. 1.**
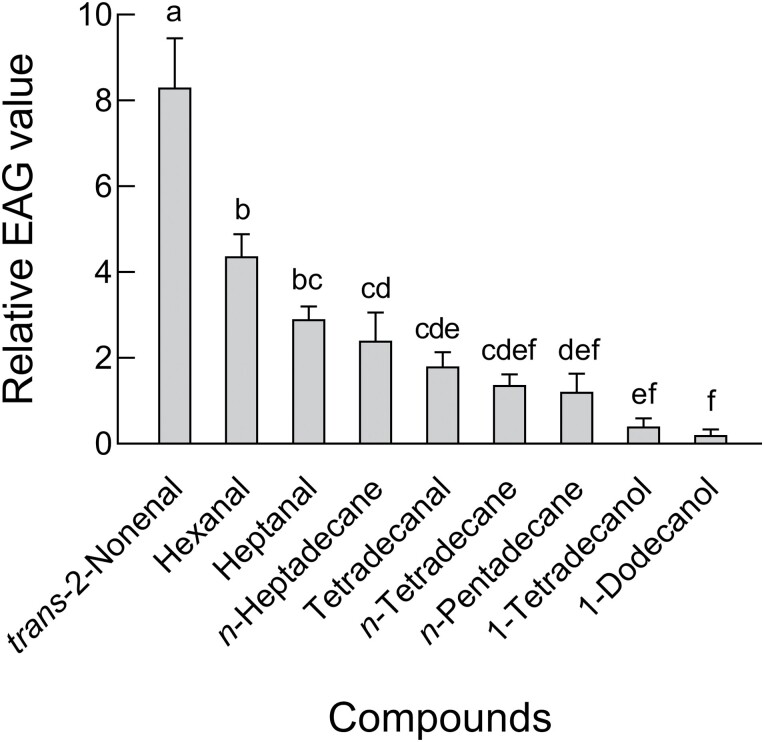
Relative EAG response (mean ± SE, *n* = 3) of female *Tamarixia radiata* antenna to 20 μl of standard substance at 10 μg/μl. Mean EAG responses to the applied concentration of the same standard substance were compared by ANOVA and LSD test (*F* = 22.39, *df*_1_ = 8, *df*_2_ = 18, *P* < 0.001). Significant differences are indicated by different letters.

Antennal responses to the 9 commercially available compounds were tested using EAG. Mean EAG responses to the applied concentration of the same standard substance varied significantly among the compounds tested (*F* = 22.39, *df*_1_ = 8, *df*_2_ = 18, *P* < 0.001). When comparing these compounds at a concentration of 10 μg/μl to the *cis-*3-hexen-1-ol (used as a control), *trans-*2-nonenal exhibited the highest relative EAG value, reaching 8.3. Following that, hexanal showed a relative EAG value of 4.3, heptanal had a value of 2.9, *n-*heptadecane measured 2.4, tetradecanal had a value of 1.8, *n-*tetradecane measured 1.4, *n-*pentadecane showed a value of 1.2, and 1-tetradecanol had the relative EAG value of 0.4. Among the tested compounds, 1-dodecanol induced the lowest relative EAG value, which was only 0.2 (Fig. 1). Based on these results, we selected the top 5 compounds for further EAG dose–response experiments.

**Fig. 2.**
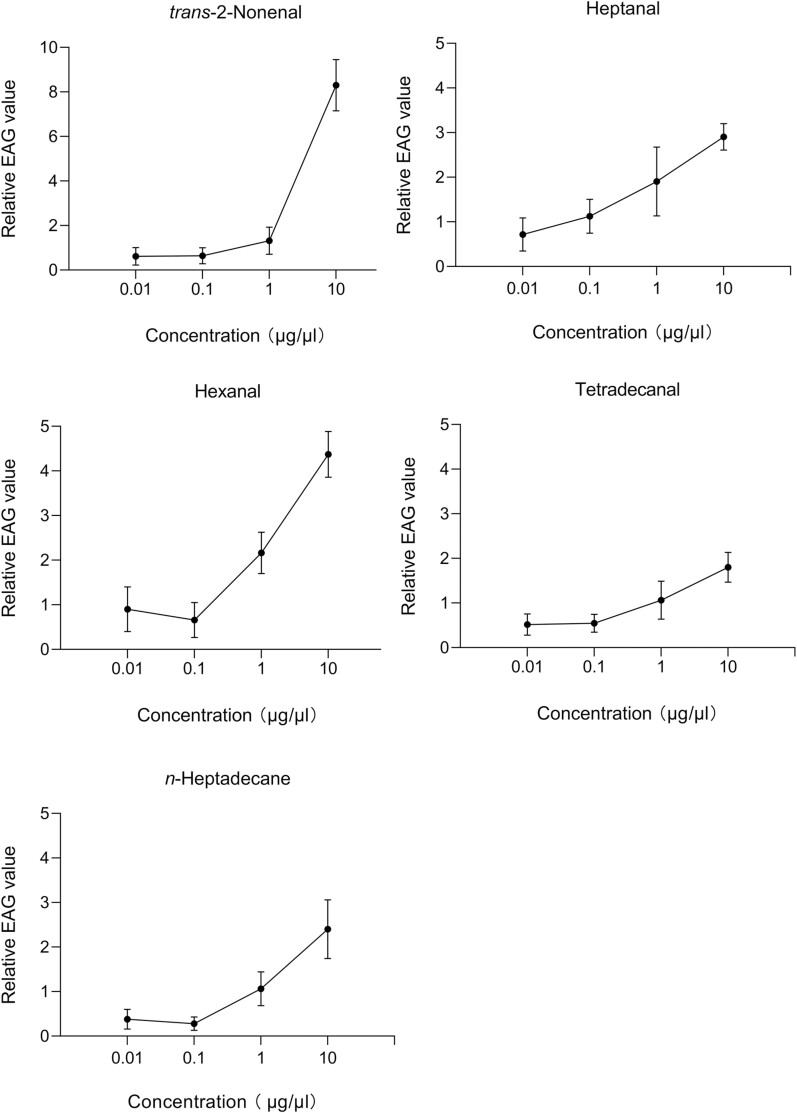
EAG concentration–response curves of female *Tamarixia radiata* antenna to *trans*-2-nonenal, heptanal, hexanal, tetradecanal, and *n*-heptadecane (mean ± SE, *n* = 6). Data represent means of triplicate identical replicates (*n* = 3); error bars depict standard deviations of means.

The EAG results of these 5 selected compounds also demonstrated positive dose–responses, as the concentrations increased from 0.01 to 10 μg/μl. Notably, for hexanal and *n-*heptadecane, the concentration of 0.1 μg/μl induced the lowest EAG response compared to the other concentrations (Fig. 2). Alternatively, the amplitude of the EAG response in the antennae for the remaining 3 compounds (*trans-*2-nonenal, heptanal, and tetradecanal) increased consistently from 0.01 to 10 μg/μl (Fig. 2).

### Olfactometer Behavior Assays

**Fig. 3.**
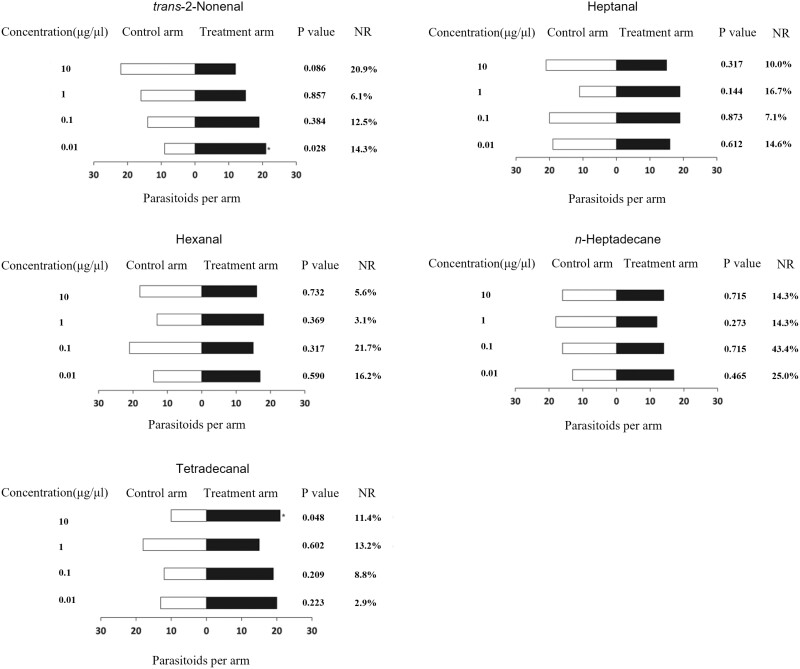
Response of female *Tamarixia radiata* to *trans*-2-nonenal, heptanal, hexanal, *n-*heptadecane, and tetradecanal vs. blank controls within a Y-tube olfactometer. * indicates significant differences between the white bars and black bars (*P < 0.05). NR: percentage of nonresponders.

To assess the biological activities of the compounds, the individual female wasps were introduced into the Y-tube olfactometer to analyze the behavioral response. This demonstrated that female adults exhibited a significant attraction to tetradecanal at the higher concentration of 10 µg/µl (*χ*^2^ = 3.9, *P* = 0.048), while no response was observed at the lower concentrations (Fig. 3). However, female *T. radiata* were attracted to *trans-*2-nonenal at 0.01 µg/µl (*χ*^2^ = 4.8, *P* = 0.028) (Fig. 3). There was no significant dose–response attraction of adults female *T. radiata* to hexanal, heptanal, and *n-*heptadecane (Fig. 3).

## Discussion

It is well-documented that parasitoids and predators use olfactory cues to locate their pest hosts. Over long distances, they can utilize plant or pest-induced volatiles to narrow down the search area. Subsequently, volatiles emitted directly from the pest or its secretions serve as short-distance olfactory cues, enhancing the success of foraging efforts (Turlings and Wäckers 2004). *D. citri* is an important pest in citrus production all over the world. Biological control has been identified as an effective strategy to manage this pest while also curbing the spread of HLB disease. *T. radiata*, a wasp species, play a crucial role in reducing the population of *D. citri* by parasitizing the later nymphal stages. Understanding the volatile compounds emitted by *D. citri* nymphs is essential for gaining insights into how parasitoids, including *T. radiata,* utilize their host insects. In this study, we analyzed the volatile components released by *D. citri* nymphs and employed electrophysiology and behavioral methods to identify the activity of these volatile compounds on female *T. radiata.* Our results showed that 9 scent compounds were preliminarily identified from *D. citri* nymphs by GC–MS when compared with blank controls. Then, the EAG and Y-tube experiments provided compelling evidence that tetradecanal and *trans-*2-nonenal significantly attracted female *T. radiata* toward the source. These compounds are likely to facilitate the swift and accurate location of the host (*D. citri*) by *T. radiata*.

The EAG technique is an electrophysiology method widely used to study antennal responses of insects to semiochemicals, allowing direct detection of their chemical signal perception (Tian et al. 2011). However, it is important to note that EAG represents the collective response of multiple olfactory receptors, synchronously reacting to the stimulus (Dickens et al. 1985). In the present study, the results indicated that female *T. radiata* showed EAG responses to 9 volatile compounds (Fig. 1: hexanal, heptanal, *trans-*2-nonenal, tetradecanal, *n-*tetradecane, *n-*pentadecane, *n-*heptadecane, 1-dodecanol, and 1-tetradecanol), which were collected from fifth instar nymphs of *D. citri*. Among these compounds, *trans-*2-nonenal elicited the most potent EAG response at a concentration of 10 µg/µl, followed by hexanal, heptanal, *n-*heptadecane, tetradecanal, *n-*tetradecane, *n-*pentadecane, 1-tetradecanol, and 1-dodecanol. This variation in response might be attributed to the chemical functionality, as it can influence the amplitude of the elicited response (Burguiere et al. 2001). The EAG response of female *T. radiata* to the volatile compounds displayed differences with varying concentrations and also among specific compounds at a given concentration. Generally, the EAG response increased with concentrations from 0.01 to 10 µg/µl for hexanal, heptanal, *trans-*2-nonenal, tetradecanal, and *n-*heptadecane (Fig. 2). This phenomenon indicates that the sensitivity of female *T. radiata* to a volatile compound is concentration-dependent. The EAG response increased in a dose-dependent manner, with greater stimulus strength resulting in heightened antennal responses. Many of the 5 volatiles have been reported to elicit EAG responses in other insects. For instance, *trans-*2-nonenal was found to evoke a strong EAG response in oriental armyworm *Mythimna separata* (Walker) (Lepidoptera: Noctuidae) (Lihuang et al. 2017). Similarly, hexanal elicited significant EAG responses in female *T. radiata*, as well as in female *Tuta absoluta* (Meyrick) (Lepidoptera: Gelechiidae), a major pest of tomato (*Solanum lycopersicum* L., Solanacae) and other solanaceous plants (Anastasaki et al. 2018). Likewise, female insects have previously shown similar antennal responses to heptanal, as noted in *Microplitis croceipes* (Cresson) (Hymenoptera: Braconidae) (Li et al. 1992). In summary, the EAG results indicated female *T. radiata* could detect all tested volatile compounds, and their antennal olfactory receptors seem to exhibit considerable sensitivity to the chemical stimuli employed. Additionally, the EAG sensitivity of female *T. radiata* varied among different volatile compounds and concentrations.

Behavioral bioassays, using a Y-tube olfactometer, are essential in order to determine the specific behavior of odor stimuli. Although EAG is a very helpful tool for measuring the activity of olfactory receptor neurons, behavioral bioassays using a Y-tube olfactometer are crucial for determining the specific behavioral responses to odor stimuli, while EAG analysis is a valuable tool for measuring the activity of olfactory receptor neurons (Birch 1971). Our study recorded consistent antennal responses in female *T. radiata* toward tetradecanal and *trans-*2-nonenal through both EAG analysis and Y-tube olfactometer tests. This indicates that female *T. radiata* possesses electrophysiological capabilities to detect tetradecanal and *trans-*2-nonenal compounds emitted by *D. citri* nymphs, and then elicits a behavioral response from the parasitic wasp (Fig. 3). In the Y-tube olfactometer bioassays, female *T. radiata* exhibited significant attraction toward tetradecanal at a concentration of 10 µg/µl and *trans-*2-nonenal at 0.01 µg/µl. However, there were no significant differences in other concentrations of tetradecanal and *trans-*2-nonenal. This discrepancy may be attributed to differences in the concentration of *trans-*2-nonenal and tetradecanal used. For instance, it has been reported that *trans-*2-nonenal at 50 ppm acts as an insect repellent for the American cockroach *Periplaneta americana* (L.) (Blattodea: Blattidae) (Meloan 1984). In addition, *trans-*2-hexenal repelled *Cotesia plutellae* (Kurdjumov) (Hymenoptera: Braconidae) at a concentration of 100 µl/ml, but it attracted the insect at lower concentrations of 1 and 10 µl/ml (Yang et al. 2016). Similarly, methyl salicylate was found to be an attractant for the green lacewing, *Chrysopa nigricornis* (Burmeister) (Neuroptera: Ceratopgonidae) (James 2003), whereas Blackwell et al. (1997) reported that methyl salicylate acted as a repellent for the host-seeking parous female *Culicoides impunctatus* (Goetghebuer) (Diptera: ceratopogonidae). The results of our Y-tube olfactometer experiments indicate that female *T. radiata* are attracted to tetradecanal and *trans-*2-nonenal at specific concentrations, suggesting that these 2 compounds may serve as active semiochemicals involved in the host location and attraction of female *T. radiata* toward *D. citri* nymphs. None of the other compounds or concentrations tested significantly attracted female *T. radiata* compared to the solvent control. This suggests that hexanal, heptanal, and *n-*heptadecane may not play a major role in the host location process of *T. radiata*.

The antennal responses of female *T. radiata* to the remaining compounds (hexanal, heptanal, and *n-*heptadecane) did not consistently correlate with their behavioral reactions (Fig. 3). There was a disparity between the antennal responses of female *T. radiata* and their subsequent behavioral responses. Such incongruities between behavioral tests and EAG assay responses have been noted in previous studies (Canale et al. 2015). Similar discrepancies between behavioral and antennal responses have been documented in other insect species, including the Kudzu bug, *Megacopta cribraria* (Fabricius) (Hemiptera: Plataspidae) (Yang et al. 2019), diamondback moth, *C. plutellae* (L.) (Lepidoptera: Yponometidae) (Yang et al. 2016), and the mirid bugs *Adelphocoris suturalis* (Jakovlev) (Hemiptera: Miridae), *Adelphocoris lineolatus* (Geoze) (Hemiptera: Miridae), and *Adelphocoris fasciaticollis* Reuter (Hemiptera: Miridae) (Xiu et al. 2019). These disparities in the present study may be attributed to 2 main reasons. First, our tests involved individual insect volatile compounds. It is conceivable that a combination of other insect volatile compounds alongside hexanal, heptanal, and *n-*heptadecane might be necessary to elicit a significant response in female *T. radiata*. Similar observations have been made by Uefune et al. (2013) where a blend of *n-*heptanal, α-pinene, sabinene, and (Z)-3-hexenyl acetate attracted *Cotesia vestalis* (Haliday) (Hymenoptera: Braconidae), whereas none of these individual compounds alone elicited attraction from the parasitoid. Additionally, the predatory mite *Neoseiulus womersleyi* (Schicha) (Acari: Phytoseiidae) was attracted to a mixture of (E,E)-α-farnesene, (E)-4,8-dimethyl-1,3,7-nonatriene, and (E)-β-ocimene, while mixtures lacking any one of these chemicals failed to attract the mite (Ishiwari et al. 2007, Uefune et al. 2013). Second, these differences might arise from variations in the relative abundance of hexanal, heptanal, and *n-*heptadecane within the semiochemical composition, as demonstrated in studies involving *trans-*2-hexenal against *C. plutella* (Yang et al. 2016). Similarly, the antennal responses of female *T. radiata* to tetradecanal and *trans-*2-nonenal at other concentrations did not consistently correlate with their behavioral reactions. These differences may arise from variations in the relative abundance of tetradecanal and *trans-*2-nonenal, as demonstrated in studies involving *trans-*2-hexenal against *C. plutella* (Yang et al. 2016). This is particularly relevant given that the behavioral significance of the tested compounds cannot be directly inferred solely from the amplitude of the EAG responses elicited (Burguiere et al. 2001). In essence, the EAG technique, which gauges overall olfactory response to odor stimuli, might not provide a complete understanding of the active semiochemicals influencing female *T. radiata* (Turlings and Wäckers 2004, Ngumbi et al. 2010).

GC–MS, EAG, and olfactometer bioassays offer valuable insights into the specific compounds that influence female *T. radiata*, potentially guiding the identification of behaviorally active substances crucial for this parasitoid. This study, therefore, sheds light on the potential host-seeking volatiles employed by female *T. radiata* and paves the path toward the advancement and implementation of semiochemical-based management strategies. The volatile semiochemicals emitted by the insects, namely tetradecanal and *trans-*2-nonenal, could potentially serve as attractive substances for the development of integrated pest management approach for controlling *D. citri*. However, comprehensive field studies involving these host insect volatile compounds, whether in isolation or combination, are needed to further our understanding of their role in guiding female *T. radiata* to their host insects. Such investigations hold the promise of contributing to the enhancement of more efficacious strategies for managing *D. citri* in real-world field conditions.

## References

[CIT0001] Anastasaki E , Drizou F, Milonas PG. Electrophysiological and oviposition responses of *Tuta absoluta* females to herbivore-induced volatiles in tomato plants. J Chem Ecol. 2018:44(3):288–298. 10.1007/s10886-018-0929-1 29404818

[CIT0002] Birch MC. Intrinsic limitations in the use of electroantennograms to bioassay male pheromones in Lepidoptera. Nature. 1971:233(5314):57–58. 10.1038/233057a0 16063193

[CIT0003] Blackwell A , Wadhams LJ, Mordue W. Electrophysiological and behavioural studies of the biting midge, *Culicoides impunctatus* Goetghebuer (Diptera, Ceratopogonidae): interactions between some plant-derived repellent compounds and a host-odour attractant, 1-octen-3-ol. Physiol Entomol. 1997:22(2):102–108. 10.1111/j.1365-3032.1997.tb01146.x

[CIT0004] Bové J , Barros A. Huanglongbing: a destructive, newly emerging, century-old disease of citrus. J Plant Pathol. 2006:88(1):7–37. http://www.jstor.org/stable/41998278

[CIT0005] Burguiere L , Marion-Poll F, Cork A. Electrophysiological responses of female *Helicoverpa armigera* (Hübner) (Lepidoptera: Noctuidae) to synthetic host odours. J Insect Physiol. 2001:47(4-5):509–514. 10.1016/s0022-1910(00)00119-0 11166315

[CIT0006] Canale A , Benelli G, Germinara GS, Fusini G, Romano D, Rapalini F, Desneux N, Rotundo G, Raspi A, Carpita A. Behavioural and electrophysiological responses to overlooked female pheromone components in the olive fruit fly, *Bactrocera oleae* (Diptera: Tephritidae). Chemoecology. 2015:25(3):147–157. 10.1007/s00049-014-0183-0

[CIT0007] Chen X , Wong S, Stansly P. Oviposition behaviour of *Tamarixia radiata*: effects of host density and exposure time. Ecol Entomol. 2017:43(1):55–59. 10.1111/een.12470

[CIT0008] Dickens JC , Payne TL, Ryker LC, Rudinsky JA. Multiple acceptors for pheromonal enantiomers on single olfactory cells in the Douglas-fir beetle, *Dendroctonus pseudotsugae* Hopk. (Coleoptera: Scolytidae). J Chem Ecol. 1985:11(10):1359–1370. 10.1007/BF01012137 24311179

[CIT0009] Gebiola M , Gomez-Marco F, Simmons GS, Stouthamer R. Effect of host feeding on life history traits of *Tamarixia radiata*, parasitoid of the Asian citrus psyllid, *Diaphorina citri*. BioControl. 2018:63(6):763–771. 10.1007/s10526-018-9903-7

[CIT0010] Hajeri S , Killiny N, El-Mohtar C, Dawson WO, Gowda S. Citrus tristeza virus-based RNAi in citrus plants induces gene silencing in *Diaphorina citri*, a phloem-sap sucking insect vector of citrus greening disease (Huanglongbing). J Biotechnol. 2014:176(1):42–49. 10.1016/j.jbiotec.2014.02.010 24572372

[CIT0011] Halbert S , Keremane M. Asian citrus psyllids (Sternorrhyncha: Psyllidae) and greening disease of citrus: a literature review and assessment of risk in Florida. Fla Entomol. 2004:87(3):330–353. 10.1653/0015-4040(2004)087[0330:acpspa]2.0.co;2

[CIT0012] Hall DG , Richardson ML, Ammar E-D, Halbert SE. Asian citrus psyllid, *Diaphorina citri*, vector of citrus Huanglongbing disease. Entomol Exp Appl. 2013:146(2):207–223. 10.1111/eea.12025

[CIT0013] Hawkings C , Morgan KJ, Shaffer LJ, Powell CA, Borovsky D, Cave RD, Dawson B, Gowda S, Shatters RG. RNAi-based strategy for Asian citrus psyllid (*Diaphorina citri*) control: a method to reduce the spread of citrus greening disease. J Citrus Pathol. 2014:1(1):160. 10.5070/C411025079

[CIT0014] Hoddle M , Stosic C, Triapitsyn S, Khan S, Arif M. How many primary parasitoid species attack nymphs of *Diaphorina citri* (Hemiptera: Liviidae) in Punjab, Pakistan? Fla Entomol. 2014:97(4):1825–1828. 10.1653/024.097.0459

[CIT0015] Ishiwari H , Suzuki T, Maeda T. Essential compounds in herbivore-induced plant volatiles that attract the predatory mite *Neoseiulus womersleyi*. J Chem Ecol. 2007:33(9):1670–1681. 10.1007/s10886-007-9344-8 17786519

[CIT0016] James DG. Field evaluation of herbivore-induced plant volatiles as attractants for beneficial insects: methyl salicylate and the green lacewing, *Chrysopa nigricornis*. J Chem Ecol. 2003:29(7):1601–1609. 10.1023/a:1024270713493 12921438

[CIT0017] Li Y , Dickens J, Steiner W. Antennal olfactory responsiveness of *Microplitis croceipes* (Hymenoptera: Braconidae) to cotton plant volatiles. J Chem Ecol. 1992:18(10):1761–1773. 10.1007/BF02751101 24254718

[CIT0018] Li Y , Zhou Y, Guo C, Ou D, Qureshi JA, Sang W, Qiu B-L. Temperature and host age effects on the life history of *Tamarixia radiata*, a dominant parasitoid of citrus psyllid *Diaphorina citri*. Crop Prot. 2018:114:32–38. 10.1016/j.cropro.2018.08.004

[CIT0019] Lihuang K , Zhang Z, Kim K, Huang Q, Lei C. Antennal and behavioral responses of *Mythimna separata* (Walker) to three plant volatiles. Environ Sci Pollut Res Int. 2017:24(32):24953–24964. 10.1007/s11356-017-0140-x 28918497

[CIT0020] Liu Y , Guo S, Wang F, Zhang L-H, Guo C-F, Cuthbertson AGS, Qiu B-L, Sang W. *Tamarixia radiata* behaviour is influenced by volatiles from both plants and *Diaphorina citri* nymphs. Insects. 2019:10(5):141. 10.3390/insects10050141 31100931 PMC6572530

[CIT0021] Mann RS , Ali JG, Hermann SL, Tiwari S, Pelz-Stelinski KS, Alborn HT, Stelinski LL. Induced release of a plant-defense volatile ‘deceptively’ attracts insect vectors to plants infected with a bacterial pathogen. J Citrus Pathol. 2014:1(1):146. 10.1371/journal.ppat.1002610 PMC3310815 22457628

[CIT0022] Mann RS , Qureshi JA, Stansly PA, Stelinski LL. Behavioral response of *Tamarixia radiata* (Waterston) (Hymenoptera: Eulophidae) to volatiles emanating from *Diaphorina citri* kuwayama (Hemiptera: Psyllidae) and citrus. J Insect Behav. 2010:23(6):447–458. 10.1007/s10905-010-9228-6

[CIT0023] Martini X , Pelz-Stelinski KS, Stelinski LL. Plant pathogen-induced volatiles attract parasitoids to increase parasitism of an insect vector. Front Ecol Evol. 2014:2:1–8. 10.3389/fevo.2014.00008

[CIT0024] Milosavljević I , Schall K, Hoddle C, Morgan D, Hoddle MS. Biocontrol program targets Asian citrus psyllid in California’s urban areas. Calif Agric. 2017:71(3):169–177. 10.3733/ca.2017a0027

[CIT0025] Miranda MP , Yamamoto PT, Garcia RB, Lopes JP, Lopes JR. Thiamethoxam and imidacloprid drench applications on sweet orange nursery trees disrupt the feeding and settling behaviour of *Diaphorina citri* (Hemiptera: Liviidae). Pest Manag Sci. 2016:72(9):1785–1793. 10.1002/ps.4213 26694803

[CIT0026] Ngumbi E , Chen L, Fadamiro H. Electroantennogram (EAG) responses of *Microplitis croceipes* and *Cotesia marginiventris* and their lepidopteran hosts to a wide array of odor stimuli: correlation between EAG response and degree of host specificity? J Insect Physiol. 2010:56(9):1260–1268. 10.1016/j.jinsphys.2010.03.032 20371248

[CIT0027] Ramiaranjatovo G , Reynaud B, Jacob V. Triple electroantennography captures the range and spatial arrangement of olfactory sensory neuron response on an insect antenna. J Neurosci Methods. 2023:390:109842. 10.1016/j.jneumeth.2023.109842 36965763

[CIT0028] Rosas M , Loera-Gallardo J, López-Arroyo JI. Comparison of the chemical and natural control of Asian citrus psyllid *Diaphorina citri*. Rev Mex Cienc Agric. 2013:4(4):495–501. 10.29312/remexca.v4i4.1182

[CIT0030] Skelley LH , Hoy MA. A synchronous rearing method for the Asian citrus psyllid and its parasitoids in quarantine. Biol Control. 2004:29(1):14–23. 10.1016/s1049-9644(03)00129-4

[CIT0031] Tian H , Chen Y, Huang Y. Advances in electroantennogram (EAG) technique of insect. Fujian J Agric Sci. 2011:26(5):907. 10.19303/j.issn.1008-0384.2011.05.045

[CIT0032] Turlings T , Wäckers F. Recruitment of predators and parasitoids by herbivore-injured plants. Adv Insect Chem Ecol. 2004:2:21–75. 10.1017/CBO9780511542664.003

[CIT0033] Uefune M , Kugimiya S, Ozawa R, Takabayashi J. Parasitic wasp females are attracted to blends of host-induced plant volatiles: do qualitative and quantitative differences in the blend matter? F1000Res. 2013:2:57. 10.12688/f1000research.2-57.v2 24358892 PMC3829125

[CIT0034] Xiu C , Pan H, Liu B, Luo Z-X, Williams L, Yang Y-Z, Lu Y-H. Perception of and behavioral responses to host plant volatiles for three *Adelpohocoris* species. J Chem Ecol. 2019:45(9):779–788. 10.1007/s10886-019-01102-3 31478157

[CIT0035] Yang G , Zhang Y, Gurr GM, Vasseur L, You M-S. Electroantennogram and behavioral responses of *Cotesia plutellae* to plant volatiles. Insect Sci. 2016:23(2):245–252. 10.1111/1744-7917.12308 26711914

[CIT0036] Yang L , Hu XP, Allan SA, Alborn HT, Bernier UR. Electrophysiological and behavioral responses of the kudzu bug, *Megacopta cribraria* (Hemiptera: Plataspidae), to volatile compounds from kudzu and soybean plants. J Agric Food Chem. 2019:67(15):4177–4183. 10.1021/acs.jafc.8b06765 30920823

